# The Association between Alcohol Exposure and Self-Reported Health Status: The Effect of Separating Former and Current Drinkers

**DOI:** 10.1371/journal.pone.0055881

**Published:** 2013-02-06

**Authors:** Wenbin Liang, Tanya Chikritzhs

**Affiliations:** National Drug Research Institute, Curtin University, Perth, WesternAustralia, Australia; California Pacific Medicial Center Research Institute, United States of America

## Abstract

**Aims:**

To investigate the direction and degree of potential bias introducedto analyses of drinking and health status which exclude former drinkers from exposure groups.

Design: Pooled analysis of 14 waves (1997–2010) of the U.S. National Health Interview Survey (NHIS).

**Setting:**

General population-based study.

**Participants:**

404,462 participants, from 14 waves of the NHIS, who had knownself-reported health status and alcohol consumption status.

**Measurements:**

Self-reported health status was used as the indicatorof health. Two approaches were used to classify alcohol consumption: (i)separation of former drinkers and current drinkers, and (ii) combined former and current drinkers. The prevalence of fair/ poor health by alcohol use, gender and age with 95% confidence intervals was estimated. The difference in prevalence of fair/ poor health status for lifetime abstainers, former drinkers, current drinkers and drinkers (former drinkers and current drinkers combined) were compared using Poisson regression with robust estimations of variance.

**Findings:**

Excluding former drinkers from drinker groups exaggerates the difference in health status between abstainers and drinkers, especially for males.

**Conclusions:**

In cohort study analyses, former drinkers should be assigned to a drinking category based on their previous alcohol consumption patterns and not treated as a discrete exposure group.

## Introduction

Many cohort studies from the medical epidemiology literaturehave observed a ‘J-shape’ or U-shape associationbetween alcohol consumption and risk for various types of chronic diseasesincluding coronary heart disease [1], [2], [3],stroke [3], [4] and diabetes [5], [6].The vast majority of studies appear to indicate that abstainers have a higherrisk of these chronic conditions compared to those who regularly consume alcoholat low or moderate levels, while former drinkers and heavy drinkers have thehighest risk of all [7], [8], [9]. In moststudies, former drinkers are participants who used to drink alcohol but stoppedsometime before the beginning of a study. These former drinkers are sometimesseparated from other participants who were still consuming alcohol at baselineand treated as a distinct ex-drinker group (e.g. [10], [11]).More often, however, analysts have mixed former drinkers with lifetime abstainerswho have never consumed alcohol and/or long-term abstainers 8,12. Study participants who have been exposedto alcohol at some time during their lifetime but who are consider to be ex-drinkersaccording to a study’s parameters, are almost universally observed inepidemiological studies to have higher risks for the various chronic diseasesexamined, and thus, the term ‘sick quitters’ has (rightlyor wrongly) has appeared in the literature as a catch-all phrasefor describing them [13], [14], [15], [16], [17].

From a methodological stand point, no wide-spread proceduralconsideration has been given in the epidemiological literature in relationto the real possibility that the very act of quitting drinking may be dueto one or more of the many harmful health effects that are directly or indirectly,attributable to alcohol. For instance, in relation to tobacco use, it hasbeen very clearly established that given the same level of cumulative smokingexposure, ex-smokers have similar or higher risks of tobacco-causeddisease compared to current smokers. Indeed, Doll and colleagues emphasizedthat ex-smokers and current smokers should be combined in analysesrather than being treated as two distinct groups [18].In relation to alcohol, it is reasonable to hypothesize therefore, that ifa proportion of people stop drinking due to ill-health, whether alcohol-relatedor not, then the methodological act of separating former drinkers from currentdrinkers will ultimately bias toward selecting a healthier current drinkersample [19]. It is important, therefore, to examinewhether bias may be introduced into epidemiological studies by separatingdrinkers who have stopped drinking, from those who continue to drink. Theaim of this study was to investigate the direction and degree of potentialbias introduced to analyses of drinking and health status which exclude formerdrinkers from exposure groups, using 14 waves (1997–2010)of the U.S. National Health Interview Survey (NHIS).

## Methods

This study used combined data from 14 waves (1997–2010)of the National Health Interview Survey (NHIS) obtained from theofficial website of Integrated Health Interview Series of U.S. National HealthInterview Survey: Minnesota Population Center and State Health AccessData Assistance Center, Integrated Health Interview Series: Version5.0. Minneapolis: University of Minnesota, 2012 (http://www.ihis.us).Details of the survey sampling strategy and data collection methods have beendescribed in detail elsewhere [20], [21], [22], [23], [24].Briefly, the NHIS were nationally focused and conducted by the National Centerfor Health Statistics (NCHS), Centers for Disease Control and Prevention (CDC).They were conducted to provide comprehensive estimations of health indictorsat national level, and state stratified samples were draw from all 50 statesand the District of Columbia to ensure the samples are representative at statelevel [20], [21], [22], [23], [24].Households were the basic unit of the NHIS. For each selected household, ifthere was more than one family residing in a household, all families in thehousehold were selected. One randomly selected adult (>18yrs)was selected per family to provide information in detail regarding their healthand health-related behavior, including alcohol use in the last 12 months.In this study, self-reported health status was used as the indicatorof health. Adult health status was divided into two groups for comparison: (1)excellent, very good and (2) good, fair and poor. For classificationof alcohol consumption, two approaches were used. The first approach separatedformer drinkers and current drinkers. Participants were grouped as follows: (i)lifetime abstainer, <12 drinks in lifetime; (ii) formerdrinker, 12+ drinks in lifetime, but none in past 12 months; and (iii)current drinker, 12+ drinks in lifetime and 1+ drink(s)in the past 12 months. The second approach combined former drinkers and currentdrinkers into one ‘drinking’ group, producing two groups for comparison:lifetime abstainers, <12 drinks in lifetime; and (ii) drinkers,12+ drinks in lifetime. The surveys did not provide information regardingprevious alcohol consumption among former drinkers, therefore we were restrictedto using one level of consumption (i.e. current drinkers). Thisapproach remains valid for the aim of the current study, which is to demonstratethe potential magnitude of this bias and thereby to inform future cohort studies.

### Analysis

#### Stratified analysis

For each classification, we estimated the weighted prevalenceof fair/ poor health for the matrix defined by, alcohol use, gender andage with 95% confidence intervals. We then plotted the prevalence offair/poor health estimated by the two different approaches to classifyingformer alcohol users. In addition, to illustrate the effect that mixing formerdrinkers with lifetime abstainers has on estimates of fair/ poor healthstatus, we plotted the weighted prevalence of fair/ poor health for thematrix defined by alcohol use, gender and age with 95% confidence intervals.Given that 14 waves of surveys have been used, the sampling weights (providedin the original data) were adjusted so that each wave would have an equivalentweight in the analyses.

#### Multivariate analysis

The difference in prevalence of fair/ poor health statusacross lifetime abstainers and former drinkers, current drinkers and drinkers (formerdrinkers and current drinkers combined) were compared using Poisson regressionwith robust estimations of variance. In order to include two different classificationsof alcohol use in the same model, a random sample consisting of 50%of the former drinkers and 50% of the current drinkers was selectedand regrouped into the ‘drinker’ group. There were therefore fourgroups in the model: lifetime abstainers, former drinkers (50%of all former drinkers), current drinkers (50% of all currentdrinkers) and drinkers (the other 50% of former drinkersand the other 50% of all current drinkers). The multivariate analysiscontrolled for age, gender, year of survey, marital status, highest educationalattainment, employment status in the past 1–2 weeks, family income comparedto the poverty threshold and ownership status of the family home.

## Results

**Figure 1:**
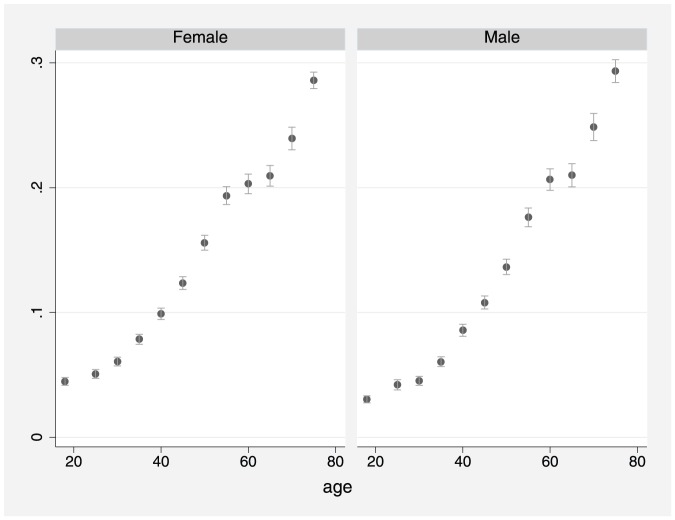
Prevalence of fair/ poor health byage for males and females (spike with caps: 95% confidenceinterval).

**Figure 2:**
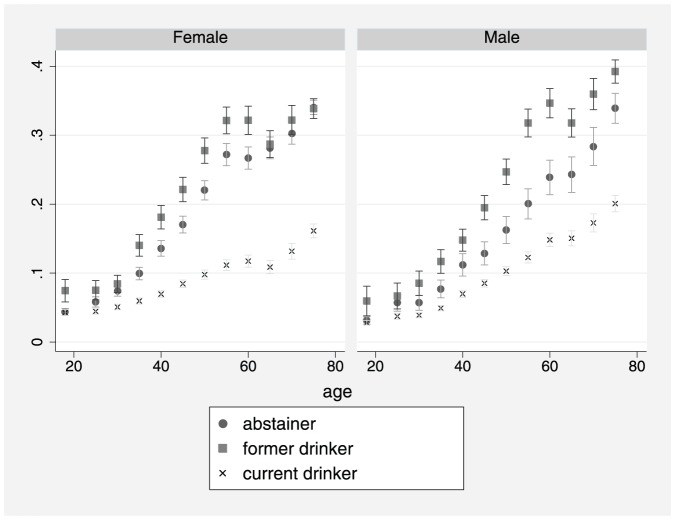
Prevalence of fair/poor health byage, gender and drinking status: Abstainers vs. formerdrinkers vs. current drinkers (spike with caps: 95%confidence interval).

**Figure 3:**
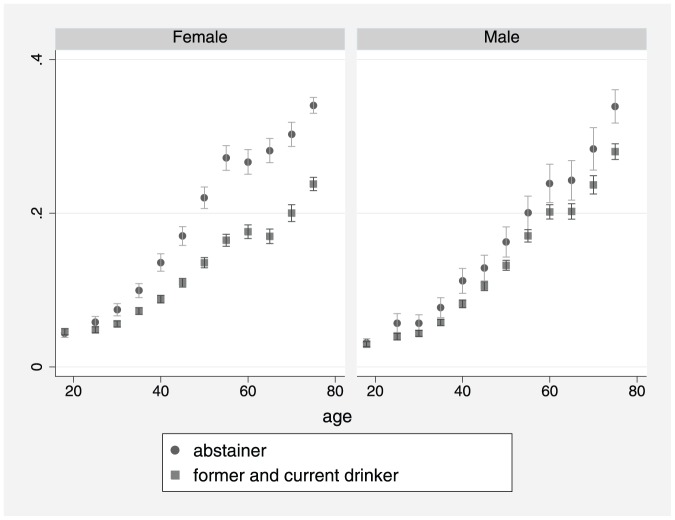
Prevalence of fair/ poor health byage, gender and drinking status: Abstainers vs. formerand current drinkers combined (spike with caps: 95% confidenceinterval).

This study included 404,462 participants, from 14 waves ofthe NHIS, who had known self-reported health status and alcohol consumptionstatus including: 97,212 lifetime abstainers (24%);62,643 former drinkers (15.5%); and 244,607 currentdrinkers (60.5%). Estimates of the prevalence of poor /fairhealth are shown in Figures 1–3. Figure 1 shows that theprevalence of poor /fair health increased with age for both males andfemales. Figure 2 shows that from about age 30yrs,former drinkers had the highest prevalence of poor/fair health, whereasthe prevalence was lowest among current drinkers, especially females. In Figure 3, former drinkers and current drinkers were pooledtogether and compared to abstainers. Among females, the difference in theprevalence of poor /fair health between abstainers and the combined drinkinggroup was reduced (i.e. compared to Figure 2)although it remained considerably large. For males, the convergence betweenabstainers and drinkers (former and current) was substantial butthe difference remained marginally significant at several ages.

Estimations from multivariate analysis (Table1) were consistent with observations from the stratified analysis.After combining former drinkers and current drinkers into a single drinkergroup for all those exposed to alcohol, the prevalence ratio of fair/poor health among drinkers compared to lifetime abstainers more closely approachedunity, especially for males.

**Table 1:** Adjusted prevalence ratio estimates of fair/poor health by drinking group (lifetime abstainers as the reference group) 1.

	Both genders			Male			Female		
	prevalence	95%	CI	prevalence	95%	CI	prevalence	95%	CI
Lifetime abstainer	1.00			1.00			1.00		
Former drinker	1.21	1.18	1.24	1.25	1.19	1.30	1.18	1.14	1.22
Current Drinker	0.75	0.73	0.78	0.81	0.77	0.84	0.72	0.69	0.74
All drinkers	0.91	0.89	0.93	0.96	0.92	1.00	0.87	0.84	0.89

1 Model controlled for age, gender, year of survey, marital status, highest educational attainment, employment status in the past 1–2 weeks, family income compared to poverty threshold and whether family home owned or rented.

## Discussion

Excluding former drinkers from drinker groups appears to exaggeratethe difference in health status between abstainers and drinkers, especiallyfor males. Fillmore et al have showed that many cohort studies had introduceda bias by mixed former drinkers with lifetime abstainers. In addition to misclassificationbias identified by Fillmore et al [12], the currentstudy demonstrated a systematic bias will still exist even after separatingformer drinkers from lifetime abstainers. These observations are consistentwith studies on the health impacts of tobacco smoking and the well-recognizedresidual health effects which impact upon the health of former smokers [18],[25],[26],[27],[28].

Given these findings, it follows that published cohort studiesof one or more chronic diseases which, compared to abstinence, find ‘protection’due to alcohol consumption among current drinkers as well as elevated risksamong those who had been exposed to alcohol at some time in the past (ex-drinkers),yet conclude protective effects, are at risk of logical incongruity. The observationthat an individual may have stopped drinking prior to the commencement ofa study does not alter the antecedent fact that they had first been exposedto alcohol.

There are lessons to be learnt here from clinical trials. Inclinical trials, it is not unusual for some participants to withdraw fromtreatment or to change their treatment plan. When this happens, results fromformer participants are preferably not separated out from the treatment groupbut are retained. This is because it has been clearly established that excluding ‘dropouts’may introduce bias which makes it appear as if the treatment group is subjectto less ill-effects or has more positive outcomes than the controlgroup. In addition, people who complete a particular treatment may, at theoutset, be predisposed to have better outcomes [29], [30].Therefore, in order to reduce bias in clinical trials, ‘intention-to-treat’analysis is recommended [30]. This essentiallyinvolves ‘returning’ any participants who had withdrawn from thetrial along with their health outcomes, back into the group which they hadoriginally been assigned prior to analysis. In the same way, for analysesundertaken on cohort studies, former drinkers should be added back to a drinkingcategory based on their previous alcohol consumption pattern.

### Conclusion

This study demonstrated that a methodological approach whichseparates past and present drinkers will likely lead to overestimation ofthe difference in health status between abstainers and drinkers, especiallyfor males. In cohort study analyses, former drinkers should be assigned toa drinking category based on their previous alcohol consumption patterns andnot treated as a discrete exposure group.
